# Isolation of *Laurus nobilis* Leaf Polyphenols: A Review on Current Techniques and Future Perspectives

**DOI:** 10.3390/foods11020235

**Published:** 2022-01-16

**Authors:** Erika Dobroslavić, Maja Repajić, Verica Dragović-Uzelac, Ivona Elez Garofulić

**Affiliations:** Faculty of Food Technology & Biotechnology, University of Zagreb, Pierottijeva 6, 10,000 Zagreb, Croatia; maja.repajic@pbf.unizg.hr (M.R.); vdragov@pbf.hr (V.D.-U.); ivona.elez@pbf.unizg.hr (I.E.G.)

**Keywords:** *Laurus nobilis* L., green extraction, conventional extraction, plant extracts, polyphenols

## Abstract

In recent years, the market demand for products enhanced with ingredients derived from natural products, such as polyphenols, is rapidly increasing. *Laurus nobilis* L., known as bay, sweet bay, bay laurel, Roman laurel or daphne is an evergreen Mediterranean shrub whose leaves have traditionally been used in cuisines and folk medicine due to their beneficial health effects, which can nowadays be scientifically explained by various biological activities of the leaf extracts. Many of these activities can be attributed to phenolic compounds present in *L. nobilis* leaves which include flavonoids, phenolic acids, tannins (proanthocyanidins) and lignans. In order to enable efficient industrial utilization of these valuable compounds, it is crucial to establish optimal extraction procedures resulting in the highest yields and quality of the extracts. This paper offers the first systematic review of current literature on the influence of conventional and advanced extraction techniques, including microwave-assisted, ultrasound-assisted, enzyme-assisted, supercritical-CO_2_ and mechanochemical-assisted extraction on the phenolic content of *L. nobilis* leaf extracts, allowing more efficient planning of further research and simplifying the steps towards industrial utilization of this plant.

## 1. Introduction

*Laurus nobilis* L., known as bay, sweet bay, bay laurel, Roman laurel or daphne is an evergreen shrub (2–20 m of height) of the Lauraceae family which includes 2500–3500 plant species that grow in the subtropics and tropics of East Asia, and South and North America [1]. The natural habitats of this plant are located in the Mediterranean area characterized by high annual precipitation [2]. Therefore, *L. nobilis* leaves have traditionally been used in Mediterranean cuisine [3] for seasoning, as well as in folk medicine along with *L. nobilis* fruits for treating viral infections, cough, rheumatism, impaired digestion, diarrhea and other health conditions [4]. Numerous scientific studies highlight the antimicrobial [5,6], antifungal [7,8], anticonvulsant [9], antioxidant [10,11,12], anti-inflammatory [13,14], antidiabetic [15,16,17], anticancer [12,18], neuroprotective [19] and anticholinergic [20] activities of *L. nobilis* leaf extracts and essential oils. These properties offer various application possibilities of *L. nobilis* extracts in the food, pharmaceutical and cosmetic industries. Due to the traditional use and commercial value of *L. nobilis* leaves, their chemical composition has been studied to a larger extent than other parts of this plant. Some of the constituents found in *L. nobilis* leaves are polyphenolic compounds, alkaloids, norisoprenoids, sugars, polysaccharides, organic acids and tocopherols [1]. The leaves also contain volatile oils which accumulate in the palisade and mesophyll cells and are present in a percentage of 1–3% on a fresh weight basis [21]. The main constituent out of around 150 identified by GC-MS in the essential oil is usually 1,8-cineol with a content ranging up to 50%, or even 70% [1,22,23]. The leaves also contain a small portion of fixed oils with 25 identified fatty acids with levels of polyunsaturated (PUFA) fatty acids higher than saturated fatty acids (SFA) and the levels of omega-3 fatty acids higher than omega-6 fatty acids, which is considered desirable for the human diet [12]. Sesquiterpene lactones also represent a characteristic group of phytochemicals present in *L. nobilis* leaves. These compounds have been reported to inhibit nitric oxide (NO) production [14] and ethanol absorption [24], as well as to increase the activity of hepatic glutathione S-transferase [25]. This group of phytochemicals is also considered as a possible cause of allergic contact dermatitis that may occur in contact with laurel leaves [26]. One of the most significant groups of bioactive compounds in *L. nobilis* leaves are polyphenolic compounds that will be more thoroughly discussed later. The total content of phenolic compounds (TPC) in laurel leaves has been reported to range from 53 to 9200 mg of gallic acid equivalent (GAE) 100 g^−1^ of extract, depending on the extraction method used [1]. Considering that the extraction of bioactive compounds from plant material is the first and crucial step in their industrial utilization, and the connection between biological activities and phenolic content of plant extracts is well-explored [27], it is of great importance to summarize the knowledge on the effects of different extraction techniques and the applied parameters on the TPC of the extracts in order to allow more directed research planning. Since, to our knowledge, no review discussing the aforementioned effects for the *L. nobilis* L. leaf polyphenols has been published, the aim of this paper was to summarize the current knowledge on the influence of different extraction techniques on the polyphenolic content of *L. nobilis* leaf extracts through a detailed search of the available literature and to propose future research possibilities.

## 2. Phenolic Compounds in *L. nobilis* Leaves

**Figure 1:**
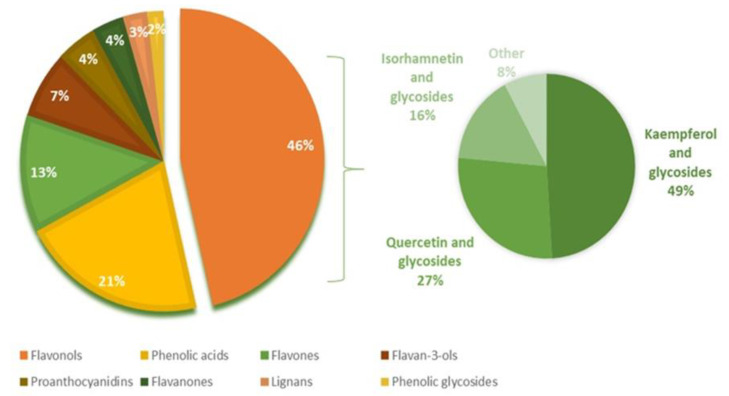
Phenolic compounds found in *L. nobilis* leaves (according to Alejo-Armijo et al. [1]; Diaz-Maroto et al. [22] and Zhilyakova et al. [28]).

*L. nobilis* leaves are a source of numerous different phenolic compounds that include flavonoids, phenolic acids, tannins (proanthocyanidins) and lignans [1]. Figure 1 shows an average composition of phenolic compounds that have been detected in *L. nobilis* leaves to date [1,22,28].

**Figure 2:**
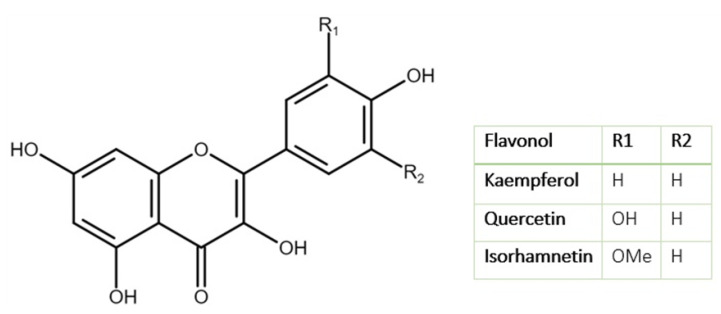
Chemical structure of the main flavonols found in *L. nobilis* L. leaves (adapted from Li et al. [29]).

As can be seen, flavonoids present the main constituents of alcoholic leaf extracts with a variety of detected compounds (Figure 1). Flavonols are present in the highest amount, with kaempferol and its glycosides being the main representatives (almost 50%), followed by quercetin and isorhamnetin and their glycosides, which are also present in significant amounts. The basic structure of the main *L. nobilis* flavonols is shown in Figure 2.

Kaempferol glycosides from *L. nobilis* have shown a variety of biological activities, such as an inhibition of NO production in lipopolysaccharide (LPS)-activated murine macrophages (J774) [30], inhibition of sodium-potassium adenosine triphosphatase [31], antioxidant activity [32], antibacterial activity against *Staphylococcus aureus, Bacillus subtilis, Micrococcus luteus, Salmonella typhimurium* and *Proteus vulgaris* [31], as well as methicillin-resistant *Staphylococcus aureus* (MRSA) and vancomycin-resistant enterococci [33]. Followed by these findings, kaempferol glycosides from *L. nobilis* leaves are especially interesting for further research focused on extraction methods that would result in their highest yield and quality. Phenolic acids from *L. nobilis* leaves have also shown antioxidant activity [34,35], and more than 20 of them have been detected [1]. Levels of *p*-coumaric and ferulic acid detected in hydroalcoholic laurel leaf extracts seem to be higher than in other herbs with similar biological potential [27]. Most of the flavones present in *L. nobilis* leaves are apigenin and its glycosides [1,23]. In a study by Al-Samarrai et al. [36] who investigated the effect of flavonoids and glycosides isolated from *L. nobilis* leaves on the lipid profile of female rabbits, apigenin-7-glucoside and luteolin-7-*O*-glucoside reduced the levels of total cholesterol and triglycerides. Tannins (proanthocyanidins) of the *L. nobilis* plant are mostly present in wooden parts [1]; however, a few, mostly lacking in structure elucidation, have also been detected in the leaves [12,37]. Cinnamtannin B-1 detected in the leaves was reported to show antioxidant activity [32].

## 3. Extraction of Phenolic Compounds from *L. nobilis* Leaves

### 3.1. Preextraction Sample Preparation

The first step in any plant extraction process is the preparation of plant samples and protection of the target compounds from deterioration. Phenolic compounds can be extracted from fresh, dried or frozen plant material. Flavonoids, particularly glycosides, which are abundant in *L. nobilis* leaves, can be degraded by intact enzymes when the plant material is fresh and undried [38]. It has been reported that the time between harvest and experimental work should be limited to 3 h in order to maintain the freshness of samples [39]. For this reason, dried and frozen plant material is usually preferred for the extraction of bioactive compounds. Plant material can be dried using several methods that include air-, oven-, microwave-, and freeze-drying (lyophilization). Air-drying at ambient temperature for a period ranging from 36 h [40] up to a few months or even a year [41], depending on the plant material, is the most preferred method since no special equipment is needed, followed by lyophilization, which is often chosen despite its complexity due to the fact that it often results in higher TPC of the final extracts [42,43]. In contrary, Papageorgiou et al. [35] have reported higher TPC and total flavonoid content (TFC) in air-dried as opposed to freeze-dried *L. nobilis* leaf extracts. Microwave- and oven-drying can cause degradation of thermolabile compounds depending on the used parameters, which could influence the final extract quality and composition. However, oven-drying at 60 °C for 48 h resulted in similar TPC as air-drying prior to heat-reflux extraction of *L. nobilis* leaves performed using the same extraction parameters [11,44]. Generally, air-drying has been the most frequently used drying method of *L. nobilis* leaves for phenolic compounds extraction, regardless of the implemented extraction method (See Section 3.2.1 and Section 3.2.2).

Drying is usually followed by milling, grinding and homogenization of the plant samples which are carried out in order to lower particle size and to increase surface contact between the sample and extraction solvent [42]. A particle size less than 500 µm is considered as the most suitable for efficient extraction [45]. Scientific data show that *L. nobilis* leaf samples were mostly ground into fine powder prior to extractions of phenolic compounds, and the size of the particles, if reported, ranged between 250–800 µm [12,45,46,47]. The presence of non-phenolic substances, such as lipids and proteins in plant material, can affect the composition and activities of phenolic compounds in the final extracts [48], thus, different purification and fractionation procedures can be applied on the crude extracts when the research is focused on composition analysis and quantification of the constituents [49]. *L. nobilis* leaves contain only 1–1.2 g of proteins and fat in traces [50], so these procedures are most often left out. Simić et al. [51] carried out a defatting process of *L. nobilis* leaves using petroleum ether and observed that defatted methanolic extracts showed a higher inhibition of lipid peroxidase. However, the research contained no data on the phenolic content, therefore the result could have been influenced by different factors.

### 3.2. Extraction Techniques

Extraction is the crucial step in isolation, analysis and utilization of phenolic compounds. Unsuitable extraction conditions may result in a lower yield of phenolic compounds or cause structural changes that would lead to undesirable effects on their biological activity [49]. The choice of the extraction procedure depends on various factors including the goal of conducted research, and nature of the plant material and target compounds. Currently, extraction processes of phenolic compounds can be divided into two groups: conventional and advanced extraction techniques [52]. Subsequent sections give a review on both groups of extraction techniques and discuss their efficacy in obtaining high TPC and TFC from *L. nobilis* leaves.

#### 3.2.1. Conventional Techniques

Conventional extraction techniques, such as infusion, decoction, digestion, maceration, and percolation, as well as Soxhlet and reflux, include the use of solvent. They are, due to their wide applicability and no special equipment requirements, the most commonly used procedures for obtaining extracts from plant material. Plant material usually contains various phenolic compounds in different quantities, ranging from simple to highly polymerized substances that may also be conjoined with other components, such as proteins and carbohydrates [53]. Therefore, an individual and systematic approach is needed to select suitable extraction parameters for every plant sample. The yield of chemical extraction depends on several parameters, including the type of solvent, solid–liquid ratio, the number of repeated extractions, stirring, extraction time, and temperature, as well as the chemical composition and physical characteristics of the plant material [54]. Parameters of conventional phenolic compounds extraction from *L. nobilis* leaves available in the literature are shown in Table 1.

##### Influence of Different Conventional Extraction Parameters on the Extraction Yield

The solvent type can affect the extraction yield of phenolic compounds due to the fact that their polarity varies between groups. For example, lower molecular flavanols and phenolic acids can be efficiently extracted using water or alcohol, such as methanol and ethanol, while polymerized procyanidins are more efficiently extracted when an aqueous solution of acetone is used [77]. Methanol, ethanol, ethyl acetate, acetone, or their combinations, often with different proportions of water, have most often been used to extract phenolic compounds from different plant material [77]. Water and hydroalcoholic mixtures of ethanol and methanol have most often been used in the extraction of *L. nobilis* leaves, as well. However, acetone was used in two studies. In the first study by Kratchanova et al. [66], the extract obtained using 80% acetone with 0.2% of formic acid after successive extraction (total time: 2 h) contained a significantly higher quantity of phenolic compounds when compared to the water extract obtained after 15 min of infusion at 90 °C. In another study [67], 15 min of infusion in boiling water resulted in higher TPC in comparison with mentioned acetone extract. This could be a result of the difference in solid–liquid ratio, which will be discussed later. The phenolic content obtained by Kratchanova et al. [66] using 80% acetone with 0.2% formic acid was similar to the one obtained by Batiha et al. [55] who used 99.5% acetone during 3 days of maceration. Since different techniques such as maceration in 80% ethanol for 5 days [57], as well as extraction in ethanol and water in a much shorter time [67] resulted in significantly higher TPC, it can be proposed that acetone is less efficient than water and hydroethanolic mixtures. However, a further comparison using the same plant material and extraction conditions would be useful in order to make valid conclusions. Other, less polar solvents, such as ethyl acetate, hexane, dichloromethane and chloroform were used in a few studies. Ethyl acetate and dichloromethane were shown to be more efficient than water, but less efficient than methanol for obtaining higher TPC [72]. Ethyl acetate was also shown to be a more efficient solvent than ethanol [59], with a more than two-fold higher TPC obtained. In the same study, use of hexane was shown to result in a slightly higher TPC than using water. As for TFC, ethyl acetate was shown to be more efficient than other non-polar solvents and water [63], but less efficient than absolute methanol [72] and ethanol [59]. According to studies which compared the efficacy of different solvents on TPC, water is a less efficient solvent than hydroalcoholic mixtures during maceration [12,37,72], as well as Soxhlet extraction [70].

Elevated temperature seems to significantly improve the efficacy of water as a solvent for extraction of *L. nobilis* phenolic compounds. Ramos et al. [62] obtained higher TPC in boiling water after 3 h, than at room temperature after 3 days. Moreover, the TPC of water extraction at 80 °C for 45 min [56] was 10-fold higher when compared to TPC obtained at room temperature during 24 h [62]. Extraction temperature and time are two significantly linked parameters, where extraction at lower temperatures requires a longer extraction time, while shorter extraction time is achieved when using moderate or high temperatures of extraction [52]. Elevated values of temperature can increase solubility of analyte and mass transfer rate, as well as decrease the viscosity and the surface tension of the solvents, which helps the solvent reach the sample matrix, resulting in an improved extraction rate. However, long extraction combined with high temperatures can increase the chance for undesirable reactions, such as hydrolysis and enzymatic oxidation of the phenolic compounds [78,79], which consequently decrease their yield in the extracts. The effect of temperature was less obvious during a comparison of TPC from different studies when other solvents were used. The highest TPC out of all conventional extraction parameters expressed as mg GAE g^−1^ extract was obtained when using 90% methanol with the addition of acetic acid (1% of volume) during 24 h maceration at room temperature [61], and it was two-fold higher than TPC obtained in 80% ethanol after 5 days of maceration at room temperature [57]. Addition of acid into organic solvent was shown to have an effect when preparing anthocyanins-rich extract because the mixture denatures the cell membranes and dissolves the anthocyanins while stabilizing them at the same time [49], so it is possible that a similar effect on other phenolic compounds of *L. nobilis* leaves enhanced the extraction yield in the mentioned study. Methanol was also reported as a more efficient solvent in comparison with ethanol and chloroform for Soxhlet extraction [70,71]. This data implies that methanol is the most efficient solvent for extraction of phenolic compounds from *L. nobilis* leaves. However, the concentration of methanol seems to have a significant effect on TPC. The TPC obtained when 50% methanol was used [34] was lower than the yields obtained by most other conventional extraction parameters. Boulila et al. [72] also reported a difference in the TPC connected to the methanol concentration. The TPC obtained in their research was higher in absolute methanol than in 80% methanol. Methanol also seems to be the most efficient solvent for obtaining higher TFC. Dhifi et al. [58] obtained more than five-fold higher TFC when using absolute methanol during a two-fold shorter extraction time than Vinha et al. [37] when using water, 50% ethanol and absolute ethanol. Due to its known toxicity, however, methanol is not suitable for research that includes organisms and animal models which often take place after the extraction processes. Since ethanol is much less toxic, it is also a more suitable extraction solvent. Therefore, it is not surprising that ethanol has been most often used for extraction of phenolic compounds from *L. nobilis* leaves.

The efficiency of ethanol as a solvent depends on the water content, and ethanol–water mixtures were shown to be more efficient than absolute ethanol [37]. Muniz-Marquez et al. [11] reported that there was no significant difference in the TPC between using 35% and 70% ethanol for heat-reflux extraction, which implies that the concentration of 35% is sufficient to obtain maximum TPC. In addition, Dobroslavić et al. [68] reported that no significant difference was observed in the TPC when 50% and 70% ethanol were used during heat-reflux extraction. In contrast to their conclusions, the highest TPC out of all included studies (expressed as mg GAE g^−1^ leaves) was the one obtained using 80% ethanol during 60 min of extraction [75]. Muniz-Marquez et al. [11] also reported that 2 h was sufficient for obtaining maximum TPC, and further extension of extraction for 8 h had no positive effect. However, the TPC obtained in a water bath shaker after 24 h in 60% ethanol [73] was four-fold higher than in the study by Muniz-Marquez et al. [11]. Moreover, the highest TPC obtained using aqueous ethanol (expressed as mg GAE g^−1^ extract) was achieved after maceration in 80% ethanol after 5 days [57]. These results might be caused by a combination of extraction parameters; however, they suggest that the extraction time cannot be excluded as an important factor for using ethanol as a solvent.

Generally, an increase of the solvent amount enhances phenolic extraction. However, it is advisable to determine an optimum ratio of the sample to solvent in order to minimize solvent input and saturation effects. Different ratios have been used in studies where bioactive compounds were extracted from plant material, and 1:12 [plant material (g): solvent (mL)] seems to be the most commonly used [52]. A ratio of 1:60 is considered sufficient for the extraction of most phenolic compounds from plant tissues [80]. In conventional extractions of phenolic compounds from *L. nobilis* leaves, a solid–liquid ratio of 1:10 and 1:20 were the most often applied. In a study where acetone was used as a solvent [55] with a resulting ratio of 2:1 after 72 h in TPC, similar to the TPC obtained when 80% acetone with 0.2% formic acid was used at a ratio of 1:40 for 2 h [66]. The difference in the extraction time indicates that the increased amount of solvent significantly influenced the extraction efficiency. At the same ratio of 1:40, water infusion at 90 °C during 15 min resulted in significantly lower TPC when compared to the acetone extract [66]. However, the extract obtained by boiling water infusion during 15 min at a ratio of 1:20 [67] resulted in significantly higher TPC than both acetone and water extract at 1:40, indicating the importance of establishing an optimum ratio for each solvent.

#### 3.2.2. Advanced Extraction Techniques

**Figure 3:**
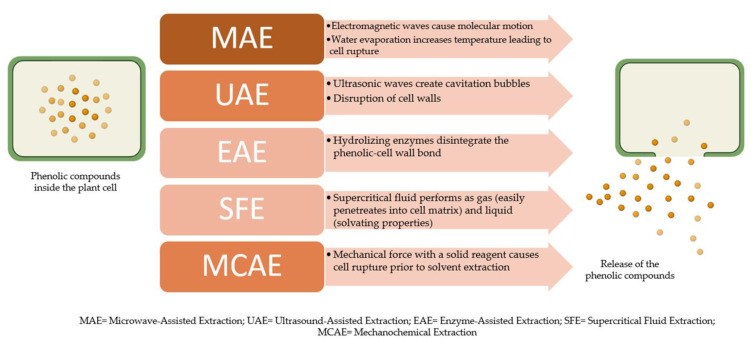
Basic principle of the advanced extraction techniques applied in *L. nobilis* leaf polyphenols isolation.

Conventional extraction techniques require a longer extraction time and large amounts of organic solvents which can cause environmental pollution. Furthermore, they have low extraction selectivity and are difficult to be automated [81]. Because of these limitations, a number of new techniques have been developed aiming to reduce organic solvent consumption and sample degradation, eliminate additional steps after the extraction, and improve overall extraction efficiency and selectivity [82]. Microwave- (MAE), ultrasound- (UAE), and enzyme-assisted (EAE) techniques, supercritical fluid extraction (SFE), and an emerging new technology called mechanochemistry have been used for the extraction of phenolic compounds from *L. nobilis* leaves, and will be further discussed. Basic principles of these techniques are shown in Figure 3. Table 2 summarizes the advantages and disadvantages of the advanced extraction techniques. Parameters from available studies on advanced extraction of phenolic compounds from *L. nobilis* leaves are shown in Table 3.

#### 3.2.3. Microwave-Assisted Extraction (MAE)

MAE is an extraction technique that uses non-ionizing radiation of electromagnetic waves with a frequency between 300 MHz to 300 GHz in order to induce molecular motion in polar or polarizable materials or solvents by working with dipoles [92]. The molecular motions result in heating of the sample, which leads to evaporation of moisture from plant cells that creates pressure, causing rupture of the cell wall and release of target compounds [93]. During radiation, the solvent molecules are induced to align themselves in a normal phase with an electric field. Under the rapid change of the electric field which occurs in MAE, solvent molecules fail to realign and start vibrating, which causes heating of the solvent due to frictional forces [53]. This allows the solvent to penetrate the plant matrix easily and promotes the extraction of the target compounds. Solvents should be chosen based on their boiling points, and dissipation and dielectric properties. Based on those properties, aqueous acetone, ethanol, or their mixtures have often been used to extract phenolic compounds using MAE [53]. Since the microwave energy is transferred by dielectric absorption only [83], non-polar solvents with lower dielectric constants can absorb much less energy, which may result in poor heating and lower extraction yields. Therefore, MAE is considered to be a selective method in the case of polar molecules and solvents with a high dielectric constant [83]. MAE has many advantages similar to UAE, including the use of less solvents, reduced extraction time and processing costs, as well as increased extraction yields. However, this technique is limited to small-molecule phenolic compounds, such as phenolic acids, quercetin, isoflavone, and trans-resveratrol, which were shown to be stable under microwave heating conditions up to 100 °C for 20 min [94]. Phenolic compounds with a higher number of hydroxyl-type substituents, such as tannins, or thermosensitive compounds, such as anthocyanins, may not be suitable for MAE. *L. nobilis* leaves, as described earlier in the text, are abundant in small-molecule flavonoids and phenolic acids, which makes MAE a suitable technique for their extraction.

This technique was previously used in three studies [44,68,70], where phenolic compounds were extracted from *L. nobilis* leaves. In all of them, aqueous solutions of ethanol in different concentrations were used as the solvent. Muniz-Marquez et al. [44] reported that ethanol concentration was the most significant influencing factor for TPC, contrary to the results reported by Dobroslavić et al. [68] where ethanol concentration had no significant influence on the TPC. At lower ethanol concentrations, Muniz-Marquez et al. [44] reported that irradiation time had very little effect on yield, while at a concentration of 50%, the TPC increased proportionally with prolonged irradiation time. The highest TPC was achieved after 9 min, and it was two-fold lower than the TPC obtained by Rincon et al. [70] after 60 min when using pure ethanol as solvent. However, in their study, the TPC after 15–30 min was lower than the yield that Muniz-Marquez et al. [44] achieved after 6 min with 50% ethanol. This indicates that use of 50% ethanol under MAE conditions of Muniz-Marquez et al. [44] is more time-efficient, which can be substantiated by results recently reported by Dobroslavić et al. [68] where 10 min was optimal during the extraction or *L. nobilis* leaf polyphenols with 50% ethanol. The presence of water in ethanol increases the dielectric constant of the system, which could result in an increased extraction yield by improving the swelling of the plant material and therefore increasing the surface contact of the matrix and solvent [95,96]. Moreover, a high ethanol concentration might interrupt the extraction of some phenolic compounds due to lower solubility and lower penetration of ethanol into the plant matrix [97]. The influence of the irradiation power and temperature must not be excluded, since Rincon et al. [70] performed the extraction at 90 °C and 500 W, which might have caused degradation of thermosensitive phenolic compounds over a prolonged time. In accordance, Dobroslavić et al. [68] reported that the increase of temperature from 40 to 80 °C resulted in higher TPC; however, with an irradiation time prolonged from 10 to 15 min, a stagnation of the TPC was observed, which was brought by the authors into connection with possible thermal degradation. The authors have also observed a decline in the TPC when an irradiation power higher than 400 W was applied. The results of these studies were most likely influenced by a combination of extraction parameters, so further research would be needed for better conclusions. Moreover, it would be interesting to see how other solvents would influence TPC.

#### 3.2.4. Ultrasound-Assisted Extraction (UAE)

UAE, often referred to as sonication, is a technique that uses ultrasonic waves ranging from 20 to 2000 kHz [83] in order to create cavitation bubbles near the sample tissue, which break down and disrupt cell walls. Consequently, surface contact between the sample and solvent increases, thereby improving mass transfer, which helps the target compounds to be extracted more efficiently [98]. Extract recovery is influenced by several factors, including sonication time, extraction temperature, solvent selection, solid–liquid ratio, wave frequency, and ultrasonic wave distribution [99]. Ultrasonic wave distribution is usually not uniform and the wave power decreases with an increased distance from the radiating surface, which is why agitation or shaking can be useful. The main benefits of UAE are reduction in extraction time and solvent consumption, which makes it a simple and relatively low-cost technology. In addition, a reduced processing time makes this technique suitable for the extraction of thermolabile compounds. However, ultrasound waves over 20 kHz may cause free radical formation and undesirable changes of target compounds [83].

Water, ethanol and methanol with different proportions of water have been used as solvents for the UAE of phenolic compounds from *L. nobilis* leaves. Hydroethanolic mixtures were shown to be more efficient than water [47,85] and absolute ethanol [85] for obtaining higher TPC and TFC. According to Muniz-Marquez et al. [47], 35% ethanol is sufficient for obtaining maximum TPC, while further increase of ethanol proportion results in lower yields. On the other hand, Dobroslavić et al. [68] reported a higher TPC when 70% ethanol was used. Since the shortest irradiation time in the study by Muniz-Marquez et al. [47], who applied the frequency of 40 kHz, was two-fold longer than 10 min, which was reported as optimal by Dobroslavić et al. [68] where 20 kHz ultrasonic probe was used, it is possible that the yield was influenced by the duration of exposure to high frequency (over 20 kHz), which might have caused undesirable changes to the phenolic compounds [90]. On the other hand, Rincon et al. [70] reported that the highest TPC was obtained after 2 h with frequency of 50/60 kHz. However, the TPC obtained in their study after 45 min of sonication at 50/60 kHz was two-fold lower than the one obtained after 40 min in 35% ethanol at 40 kHz [47], which could have been a result of the effect of the solvent mentioned in Section 3.2.3, as well as the frequency of ultrasonic waves. Another factor which, according to Muniz-Marquez et al. [47], significantly influences the TPC, was a solid–liquid ratio, which when decreased from 1:4 to 1:12 g of sample per mL of solvent, lead to an increased TPC. In accordance, Dobroslavić et al. [68] achieved a two-fold higher TPC by applying a solid–liquid ratio of 1:50 g of sample per mL. As for other solvents, methanol appears to be a less efficient solvent for UAE when compared to ethanol, since 2 h of extraction in absolute ethanol with a solid–liquid ratio of 1:40 [70] resulted in significantly higher TPC in comparison with 2 h of extraction in absolute methanol with a solid–liquid ratio of 1:100 [84]. However, it is possible that the difference in the solid–liquid ratio might have also influenced the results. Further research on the same plant samples would be necessary to make more valuable conclusions.

#### 3.2.5. Enzyme-Assisted Extraction (EAE)

EAE is considered as a novel and efficient technique for the extraction of numerous secondary plant metabolites with antioxidant properties [81]. It is based on the fact that these metabolites in plant matrices, including phenolic compounds, often interact with a polysaccharide-lignin complex in the cell wall by ester, hydrogen or hydrophobic bonding [86], which can sometimes make them unreachable for solvent during extraction. The addition of specific hydrolyzing enzymes, such as cellulase, α-amylase, pectinase and hemicellulase might enhance extraction of phenolic compounds by promoting disintegration of the phenolic-cell wall matrix bonds, thus allowing the entrance of solvent [87,100]. The most important factor for extraction efficiency of phenolic compounds, along with the pH of the system, extraction temperature and time, and enzyme concentration, was found to be the particle size of the samples [101]. With an increased contact surface caused by a smaller particle size, the enzyme action is increased. EAE has important shortcomings, which include high costs of required enzymes and the difficulty of applying laboratory scale conditions in industrial scale [102]. Boulila et al. [72] used enzyme pre-treatment in extraction of phenolic compounds from *L. nobilis* leaves and observed no significant difference in TPC and TFC between pre-treated methanolic extracts and control. The authors explained this with the presence of lignin in the cell walls (27.61% in *L. nobilis* leaves), which might limit the accessibility of cellulase and hemicellulase to their substrate.

#### 3.2.6. Supercritical Fluid Extraction (SFE)

SFE is a method where supercritical fluid, a substance that shares physical properties of both gas and liquid above its critical point [103], is used. These properties allow the performance of gas in terms of penetration power into the cell matrix, as well as the solvating properties of liquid [104,105]. CO_2_, with a critical point above 31.1 °C and 7380 kPa, is the most frequently utilized supercritical fluid in SFE. It is inflammable, relatively non-toxic, chemically stable, inexpensive, and produces zero surface tension [89]. Its mild critical temperature is suitable for extraction of thermolabile compounds [106]. However, since it is non-polar, the addition of polar modifiers, such as ethanol, methanol, ethyl acetate, or acetone is recommended for the extraction of polar phenolic compounds [107]. A pressure between 50–600 bar, temperature of 20–35 °C and time of 5–180 min are considered as the parameters that result in the highest yields of phenolic compounds extracted by SFE from various plant materials [88]. SFE has many advantages over conventional extraction techniques that include lower organic solvent consumption, increased selectivity and separation of the extract, as well as reduced extraction time [108]. The main advantage of this method is its lower possibility of sample contamination by solvent impurities and avoidance of degradation and oxidation of extracted compounds, since it is performed in the absence of air and light [103]. However, the initial cost of the SFE equipment is very high [109] and the cost of applying it in an industrial scale often outweighs the technical benefits [49].

SFE has been used to extract essential oil from *L. nobilis* leaves [2,110,111], however only Santoyo et al. [46] determined the TPC in extracts obtained using this technique. Extraction parameters which are shown in Table 3 were chosen by the authors based on their previous research on rosemary (*Rosmarinus officinalis*) and oregano (*Origanum vulgare*) leaves. It was shown that the temperature and pressure of the separators had a significant effect on TPC, as well as on antioxidant activity, of which values were higher at 20 bar and 20 °C when compared to the conditions of 100 bar and 60 °C. TPC obtained from both separators is comparable to the content obtained by other extraction techniques, including advanced and conventional ones (Table 1 and Table 3). However, more data are needed to make a valid comparison of SFE with other techniques. This can be achieved by varying different extraction parameters in order to find optimal conditions for SFE of phenolic compounds from *L. nobilis* leaves, since they can differ significantly for different plant materials [49]. SFE resulted in higher antioxidant capacity of myrtle (*Myrtus communis*) extracts when compared to conventional extraction [112]. Authors put this into correlation with a higher concentration of the myricetin-*O*-glycosides (flavonol glycosides). Since, as previously mentioned, *L. nobilis* leaves are rich in flavonol glycosides, SFE could potentially result in their higher yield and antioxidant capacity as well. A study on *Ziziphus jujuba* Mill. leaves is in agreement with this hypothesis, since it showed that the SFE technique was superior to UAE for the recovery of kaempferol and quercetin glycosides, which are abundant in *L. nobilis* leaves [113].

#### 3.2.7. Mechanochemical-Assisted Extraction (MCAE)

In order to overcome the purification difficulties due to low selectivity and solvent residues after other advanced extraction techniques, an innovative technology, MCAE, has recently emerged. This technology is based on the research of physicochemical and chemical transformation of compounds caused by mechanical force, such as grinding in a ball mill [90,114]. It consists of mechanochemical processing of plant material under highly insensitive mechanical pressure in the ball mill, with a solid reagent (usually carbonated salts) prior to solvent extraction [115]. Cell walls rupture due to this process, allowing the extraction of target compounds whose water solubility is also improved [91]. This allows the use of water instead of other conventional solvents, reducing the cost of extraction and simplifying the purification process. The most commonly used reagents have been solid alkali reagents, such as NaCO, NaHCO and NaOH, depending on their alkaline strength and the chemical properties of the target compounds [116]. Some studies [91,115] have shown that MCAE results in higher flavonoid yields while being more time-efficient at lower extraction temperatures and without use of organic solvents. However, since the technique is quite novel, the influence of different extraction parameters is still inconsistent and there is a lack of complete understanding, which is essential for the scale-up process and further application [114].

Rincon et al. [70] used Na_2_CO_3_, BaCO_3_, Li_2_CO_3_, CoCO_3_, K_2_CO_3_ and CaCO_3_ in excess of 25 or 50% as solid reagents prior to *L. nobilis* leaf extraction with ethanol. The excess of 25% was shown to result in higher TPC than when 50% excess was used. Adding 25% of Li_2_CO_3_ resulted in the highest TPC; however, the value was slightly lower than the one obtained by Soxhlet extraction with ethanol in the same study. Additionally, the highest yields obtained by MAE and UAE in the same study were significantly higher than the one obtained by MCAE. It is important to note that the highest yields in MAE and UAE were obtained after 60 and 120 min, respectively, while the total extraction time in MCAE was 40 min. Since TPC from *L. nobilis* obtained by MCAE is comparable, and even higher than the TPC obtained at certain parameters of other extraction techniques, there is definitely potential for further research and optimization of the MCAE for the extraction of phenolic compounds from *L. nobilis* leaves, which could lead to higher yields and/or lower extraction costs than other techniques.

## 4. Future Perspectives

Laurel leaves, due to a wide range of structurally diverse bioactive molecules and their antioxidant, antimicrobial, anti-inflammatory, and other health beneficial properties, are an excellent base for the production of high-quality extracts with potential applications in the food, pharmaceutical, and cosmetic industries. Insights into the biopotential of laurel require new approaches in the production of plant extracts, and consequently, the use of advanced green techniques that allow the development of formulations and high value-added products with improved biological properties and actions. This paper presents a systematic review of conventional and advanced extraction techniques for the isolation of phenolic compounds from *L. nobilis* leaves, emphasizing the importance of optimization and achieving high yields of polyphenols under optimal conditions, regardless of the applied technique. It has been shown that similar total phenolic yields can be achieved by adjusting the extraction parameters of both conventional and advanced extraction techniques. Therefore, further research should be focusing on including more extraction parameters in optimization with the aim of achieving higher yields of total polyphenols and on overall extract quality, with an emphasis on isolation of target bioactive compounds, such as kaempferol glycosides which have shown diverse biological activities. To the best of the authors’ knowledge, pressurized liquid extraction (PLE), also known as accelerated solvent extraction (ASE), has not yet been applied for the extraction of phenolic compounds from *L. nobilis* leaves. Nevertheless, comparison of PLE with conventional methods [117,118,119] has shown that PLE resulted in comparable or higher yields of phenolic compounds while being time-efficient and economic, which sets a promising perspective for application of this technique on *L. nobilis* leaves.

Since phenolic compounds are prone to losing their active properties during storage, it is of great importance to preserve their bioactivity and improve their stability to make them applicable in the industry. Therefore, future research should also be focused on various encapsulation techniques that would result in more stable forms of beads or powders with required release characteristics, biocompatibility and bioavailability of the active compounds [120,121]. Investigating bioavailability for the purposes of application in functional food and supplements is extremely important, since the abundance of polyphenols does not necessarily mean the best bioavailability profile [122]. In vitro methods for the evaluation of bioavailability cannot reproduce the complex environment of human digestion that in vivo methods can; however, they are relatively fast, simple, cheap, and reproducible, allowing more efficient formulation of the products [123]. All of the mentioned steps present future perspectives and open new areas for the multidisciplinary research and development (R&D) of sustainable, efficient and economic procedures that would result in the maximum use of the great potential which *L. nobilis* leaves and their bioactive molecules hold.

## Figures and Tables

**Table 1:** Parameters used in conventional extraction techniques of phenolic compounds from *L. nobilis* leaves.

Drying Method	Extraction Parameters	Solid–liquid Ratio (g mL^−1^)	TPC ^a^	TFC ^b^	Ref.	Publication Year
Maceration						
Electric dryer at 30 °C	99.5% acetone; 72 h; 30 °C	2:1	71.2 ± 2.5 mg GAE ^c^ g^−1^ extract	39.2 ± 7.4 mg CAE ^d^ g^−1^ extract	[55]	2020
Oven dried at 60 °C for a week	distilled water; 45′; 80 °C	1:10	137.0 mg PE ^e^ g^−1^ sample	604.12 mg CAE g^−1^ sample	[56]	2020
Air-drying	80% ethanol; 5 days; room temperature	1:5	110.43 mg GAE g^−1^ extract	-	[57]	2019
Air-drying	absolute methanol; 30′; room temperature	1:10	-	149.2 ± 8.3 mg ECE ^f^ g^−1^ extract	[58]	2018
Air-drying	hexane/ethyl acetate/ethanol/water 5× in 24 h; room temp.	-	11.04–54.42 mg PE ^f^ g^−1^ sample	1.01–8.60 mg QE ^g^ g^−1^ extract	[59]	2017
Unspecified	80% ethanol; 48 h; room temperature; Successive 24 h; evaporation at 40 °C Defatting: petroleum ether 2× Lyophilization Ethyl acetate; 20% ammonium sulphate; 2% ortho-phosporic acid	1:1001:50	25.70 mg GAE g^−1^ extract	12.11 mg QE g^−1^ extract	[60]	2016
Air-drying	90% methanol + acetic acid at 24 °C for 24 h	1:10	288.15 ± 1.34 mg GAE g^−1^ extract	-	[61]	2016
Air-drying	99% ethanol/deionized water; 3 days; room temperature deionized boiling water; 3 h	1:10	53–132 mg GAE g^−1^ extract	-	[62]	2012
Air-drying	70% methanol 3× in 24 h Ether/chloroform/ethyl acetate/*n*-butanol until colorless	1:20	-	0.68–1.56 mg g^−1^ extract	[63]	2010
Unspecified	70% ethanol, 3× 48 h	-	201 mg g^−1^ leaves	-	[64]	2006
Infusion						
Unspecified	DMSO	-	44.07 mg GAE g^−1^	60.56 mg NAE ^h^ g^−1^	[65]	2021
Air-drying	methanol; 2 × 1 h; 25 °C at 150 rpm/boiling distilled water (100 °C), 5′; room temperature	1:301:200	76.16 ± 0.34 mg g^−1^ extract/64.77 ± 2.14 mg g^−1^ extract	-	[12]	2014
Unspecified	water; 15 min; 90 °C centrifuge 6000 rpm	1:40	17.66 mg GAE g^−1^ extract	-	[66]	2010
Air-drying	boiling water (100 °C); 15′	1:8	1.03 ± 0.04 mg GAE L^−1^ infusion	-	[32]	2009
Air-drying	boiling distilled water; 15′/ ethanol; reextracted until colorless	1:20	81.7 mg GAE g^−1^ extract/ 84.5 mg GAE g^−1^ extract	-	[67]	2006
Heat-reflux extraction						
Unspecified	50–70% ethanol	1:50	42.21−42.35 mg GAE g^−1^ leaves	-	[68]	2021
Oven dried at 60 °C for 48 h	35% ethanol; 2 h; 60 °C	1:4	2.34 ± 0.93 mg GAE g^−1^ dry leaves	-	[44]	2018
Unspecified	ethanol water	1:7.5	94.07 mg GAE g^−1^ extract 66.70 mg GAE g^−1^ extract	-	[69]	2015
Air-drying	ethanol (0, 35, 70%); 0–8 h; 60 °C	1:4	1.5–10.23 mg GAE g^−1^ leaves	-	[11]	2014
Soxhlet extraction						
Oven dried at 55 °C until moisture level < 10%	water/methanol/ethanol 5 h	1:40	30.73–83.41 mg GAE g^−1^ extract 10.42–12.59 mg GAE g^−1^ dry leaves	-	[70]	2019
Air-drying	chloroform/ methanol	-	0.36 ± 0.01 mg L^−1^ extract/ 0.90 ± 0.06 mg L^−1^ extract	-	[71]	2011
Water bath shaker						
Oven dried hydrodistilled residues (temperature unspecified)	water/methanol/80% methanol/ethyl acetate/dichloromethane 48 h; 150 rpm shaker; 2× (water 1×)	1:20	0.50–5.87 mg GAE g^−1^ extract	0.15–5.18 mg QE g^−1^ extract	[72]	2015
Air-drying	60% ethanol; 24 h; 35 °C	1:20	46.79 ± 3.22 mg GAE g^−1^ dry leaves	-	[73]	2011
Centrifuge						
Oven dried at 25 ± 2 °C for 3 weeks	water/50% ethanol/ethanol 1 h, 40 °C at 600 rpm	1:10	14.37–43.03 mg GAE g^−1^ extract	14.12–30.15 mg ECE g^−1^ extract	[37]	2015
Freezed fresh leaves	phosphate buffer (75 mM, pH 7.0) 20 min; 20,000 rpm	1:7.5	4.02 mg GAE g^−1^ leaves	-	[74]	2001
Solid–liquid extraction						
Unspecified	80% ethanol; 60 min; 60 °C	1:50	148.3 mg GAE g^−1^ leaves	110.5 mg GAE g^−1^ leaves	[75]	2019
Unspecified	water; 50 °C	-	59.85 mg GAE g^−1^ leaves	-	[76]	2009
Orbital shaker						
Unspecified	80% acetone with 0.2% formic acid; 1 h; room temperature (2× successive) centrifuge 6000 rpm	1:40	70.81 mg GAE g^−1^ extract	-	[66]	2010

^a^ Total phenolic content; ^b^ Total flavonoid content; ^c^ Gallic acid equivalents; ^d^ Catechin equivalents; ^e^ Pyrocatechol equivalents; ^f^ Epicatechin equivalents; ^g^ Quercetin equivalents; ^h^ Naringin equivalents.

**Table 2:** Summary of the advanced extraction techniques applied for the extraction of *L. nobilis* L. leaf polyphenols.

Extraction Technique	Advantages Over Conventional Techniques	Disadvantages	Precautions	Number of Studies on *Laurus nobilis* L. Leaf Polyphenols Isolation	Ref.
MAE	Reduced solvent consumption Reduced extraction time Increased selectivity under right choice of solvent	Limited to small-molecule phenolic compounds	Solvents with high dielectric constant should be chosen	3	[41,44,68,70,83]
UAE	Reduced solvent consumption Reduced extraction time Low-cost technology Suitable for thermolabile compounds	Ultrasound waves over 20 kHz may cause free radical formation and undesirable changes of target compounds	The exposure time to high frequencies should be limited	7	[35,45,47,68,70,83,84,85]
EAE	Possible enhancment of the solvent permeability	High costs of required enzymes Difficulty of applying laboratory scale conditions in industrial scale	The composition of plant material might limit the access of enzymes	1	[72,86,87]
SFE	Lower possibility of sample contamination by solvent impurities Air- and light-free (avoidance of degradation and oxidation of extracted compounds)	High initial cost of the SFE equipment High cost of industrial scale application	Addition of polar modifiers recommended for phenolic compounds	1	[46,88,89]
MCAE	Water can be used as solvent (increased solubility) Reduced cost Simplified purification processes	Inconsistent data due to novelty of the technique	Solid reagents should be chosen depending on their alkaline strength and the chemical properties of the target compounds	1	[68,90,91]

MAE = microwave-assisted extraction; UAE = ultrasound-assisted extraction; EAE = enzyme-assisted extraction; SFE = supercritical fluid extraction; MCAE = mechanochemical extraction.

**Table 3:** Parameters used in advanced extraction techniques of phenolic compounds from *L. nobilis* leaves.

Drying Method	Extraction Parameters	Solid–liquid Ratio (g mL^−1^)	TPC ^a^	TFC ^b^	Ref.	Publication Year
Microwave-assisted extraction						
Unspecified	50–70% ethanol; 40–80 °C; 400/800 W; 5–15 min	1:50	30.88–53.57 mg GAE ^c^ g^−1^	-	[68]	2021
Oven dried at 55 °C until moisture level <10%	ethanol, 500 W; stirring power 50% 15–75′; 90 °C	-	25.03–135.47 mg GAE g^−1^ extract 2.74–21.56 mg GAE g^−1^ dry leaves	-	[70]	2019
Oven dried at 60 °C for 48 h	60 ± 2 °C; three-stage irradiation power (800 W, 15 s; 400 W, 15 s; 200 W, 30 s). ethanol 25–50% 3,6,9′	-	1.91–10.63 mg GAE g^−1^ plant	-	[44]	2018
Ultrasound-assisted extraction						
Unspecified	50–70% ethanol; 5–15 min; 50–100% amplitude; 20 kHz	1:50	24.43–36.74 mg GAE g^−1^ leaves	-	[68]	2021
Air-drying + 45 min oven at 50 °C	ethanol/water/50% ethanol; 20′; 45 °C; 20 kHz	1:10	476.94–796.94 µg GAE g^−1^ extract	192.82–398.71 µg CAE ^d^ g^−1^ extract	[85]	2020
Oven dried at 55 °C until moisture level <10%	ethanol; 30–150′; 360 W; 50/60 kHz	1:40	44.35–164.04 mg GAE g^−1^ extract 3.33–24.77 mg GAE g^−1^ dry leaves	-	[70]	2019
Air-drying	50% ethanol + 0.1% formic acid, 5′ sonication; centrifuge: 3000× *g;* 10′; 4 °C 2×	1:5	1.12 ± 0.08 mg GAE g^−1^ extract	-	[45]	2014
Air-drying	ethanol (0,35,70%); 20–60′; room temperature; 40 kHz	1:4; 1:8; 1:12	3.52–17.32 mg GAE g^−1^ plant	-	[47]	2013
Air drying (a) Freeze drying (f): 6 h at −60 °C	70% methanol; 6 M HCl 15′ sonication + water bath reflux: 90 °C; 2 h	1:100	a: 22.90–80.30 f: 21.50–41.20 mg GAE g^−1^ extract	a: 2.90 ± 0.18 mg ECE ^e^ g^−1^ extract f: traces	[35]	2008
Unspecified	methanol; 2 h; 40 °C ultrasonic bath	1:100	99.7 mg GAE g^−1^ extract	80.1 mg kg^−1^ extract	[84]	2005
Enzyme-assisted extraction						
Oven dried (no defined temperature) hydrodistilled residues	Pretreatment: distilled water + cellulase/hemicellulase/xylanase/ternary mixture; 1 h; 40 °C methanol; 48 h; 150 rpm shaker; 2×	1:5 1:20	5.85–7.12 mg GAE g^−1^ extract	5.18–6.33 mg QE ^f^ g^−1^ extract	[72]	2015
Supercritical fluid extraction						
Air-drying	250 bar; 60 °C; 4% ethanol; 75′ 1. separator: 100 bar, 60 °C 2. separator: 20 bar, 20 °C	-	1. 51.6 ± 0.98 mg GAE g^−1^ extract 2. 87.38 ± 1.32 mg GAE g^−1^ extract	-	[46]	2006
Mechanochemical extraction						
Oven dried at 55 °C until moisture level <10%	Na_2_CO_3_, BaCO_3_, Li_2_CO_3_, CoCO_3_, K_2_CO_3_, CaCO_3_ (excess of 25 or 50%) ball mill: 400 rpm; 10′ ethanol; 20′; magnetic stirring. centrifuge: 2683.2× *g*, 10′	-	33.01–75.54 mg GAE g^−1^ extract 1.91–9.52 mg GAE g^−1^ dry leaves	-	[70]	2019

^a^ Total phenolic content; ^b^ Total flavonoid content; ^c^ Gallic acid equivalents; ^d^ Catechin equivalents; ^e^ Epicatechin equivalents; ^f^ Quercetin equivalents.

## Data Availability

Not applicable.
